# Aging in Place: Evolution of a Research Topic Whose Time Has Come

**DOI:** 10.1155/2012/120952

**Published:** 2011-11-17

**Authors:** Sarinnapha Vasunilashorn, Bernard A. Steinman, Phoebe S. Liebig, Jon Pynoos

**Affiliations:** ^1^Office of Population Research, Princeton University, Princeton, NJ 08544, USA; ^2^Center for Gerontology and Health Care Research, Brown University, Providence, RI 02912, USA; ^3^Andrus Gerontology Center, University of Southern California, Los Angeles, CA 90089, USA

## Abstract

Over the past 30 years, policy makers and professionals who provide services to older adults with chronic conditions and impairments have placed greater emphasis on conceptualizing aging in place as an attainable and worthwhile goal. Little is known, however, of the changes in how this concept has evolved in aging research. To track trends in aging in place, we examined scholarly articles published from 1980 to 2010 that included the concept in eleven academic gerontology journals. We report an increase in the absolute number and proportion of aging-in-place manuscripts published during this period, with marked growth in the 2000s. Topics related to the environment and services were the most commonly examined during 2000–2010 (35% and 31%, resp.), with a substantial increase in manuscripts pertaining to technology and health/functioning. This underscores the increase in diversity of topics that surround the concept of aging-in-place literature in gerontological research.

## 1. Introduction

Over time, the goal of aging in place has become a focal concept by policy makers as well as researchers in their collective efforts to create communities that facilitate the widely recognized preference by a majority of older adults to remain in their homes and communities as long as possible [1–4]. Efforts to reform how and where long-term care services are provided have produced substantial programs enacted to reduce reliance on the most expensive forms of care to address disability associated with chronic disease and impairment. A shift in priorities and resources toward deinstitutionalization has resulted in explicit policies and programs that reflect a paradigm shift from nursing homes as the most likely alternative for older adults requiring multiple services to nursing homes as an option of last resort. 

Corresponding to greater policy aimed at facilitating aging in place, there has also emerged a growth in academic literature, reflecting the concerns of stakeholders (including policy makers, care providers, families, and older adults themselves), which illuminates a greater number of options aimed at stemming rising costs of care, and accommodating and facilitating the wishes of older adults to remain independent. Initial efforts to conceptualize and define aging in place as an important discussion topic focused on understanding older adults in terms of changes occurring both in themselves and in their surrounding environments. For instance, in describing the concept of environmental press,Lawton and Nahemow[5] examined dynamic interactions between housing environments and the physical capabilities of older people. In optimal settings, characteristics of the environment should function to accommodate losses of physical function. Thus, Lawton recognized the necessity of a variety of specialized living environments that could address the full range of functioning from independence to dependence on institutional care, with community housing, congregate housing, and boarding homes falling within this spectrum. Since this early seminal work, concepts of aging in place evolved to emphasize services and technology as important contributors to an older adult's ability to remain in his/her home. Indeed, Brink [6] highlighted the importance of integrating services with housing in stating that the goal of aging in place would be seriously hampered if support services are unable to keep up with their demand. Consistent with Lawton's [5] view, the primary goal of services and technology is to match the level of support provided by the housing environment to the level of capabilities (or need) of the individual. 

Over the past 30 years, policy makers and professionals who provide services to older adults with chronic conditions and impairments, as well as researchers, have placed greater emphasis on conceptualizing aging in place as an attainable and worthwhile goal. Nevertheless, there is little known of the changes over time in the attention given to aging in place within gerontological literature. With respect to the quantity and substance of the literature on aging in place, the current study was designed to provide important insight as to the prominence of environmental, service based, technology, and health factors associated with an older adult's ability, inability, or choice to age in place. Moreover, given the increasing number of older adults who express a preference to remain in their home, understanding and tracing the evolution of this topic in gerontology is more timely today than ever before. Perhaps more importantly, in studying changes in empirically based aging in place publications, light can be shed on how such temporal changes may influence policy related to services, environment, and technology. 

In our analyses, we examined the trajectory of aging in place within the context of scholarly discussions in major gerontology journals. Specifically, the purpose of our research was to examine how the literature on aging in place has changed over time in highly visible gerontology journals, with a focus on analyzing trends related to the amount, location, and variety of research topics. We hypothesize that generally there would be an increased proportion of articles dedicated to the topic of aging in place, and that among those articles, the diversity of topics covered in publications would increase over time.

## 2. Methods

In this study, we analyzed scholarly articles published from 1980 through 2010 in eleven leading gerontology journals with content areas that focus on research and/or policy pertaining to older adults. In a preliminary analysis, we examined a broad array of terms that capture the concept of aging in place. These included aging/ageing in place, aging/ageing at home, naturally occurring retirement community(s), elder friendly community(s), aging in the community, home independence, and staying put. For the terms that yielded less than 20 manuscripts within our 1980–2010 timeframe, we excluded these terms from our final search list. This left 3 critical search terms: aging/ageing in place, aging/ageing at home, and naturally occurring retirement community(s). Given that our interest was in examining trends in aging in place, we operationalized our definition of aging in place search items to include only the most commonly used terms. This approach results in a more conservative estimate of documenting trends in aging in place; nevertheless, given the minimal number of manuscripts that utilized the alternate terms pertaining to aging in place, this criteria should not substantially bias our results.

Journals were chosen based on the frequency of appearance of the 3 critical terms and their variations (aging/ageing in place, aging/ageing at home, and naturally occurring retirement community(s)) in a preliminary search using Google Scholar. If terms appeared in each journal 20 or more times during the 30-year period of interest, we included the journal in our main analysis. These journals included *Ageing & Society*; *Ageing International*; *Generations*; *The Gerontologist*; *Journal of Aging and Social Policy*; *Journal of Applied Gerontology*; *Journal of Gerontological Social Work*; *Journal of Gerontology; Journals of Gerontology Series B: Psychological Sciences and Social Sciences*; *Journal of Housing for the Elderly*; *Research on Aging*. Next, the sum total of all articles for each journal and a grand total number of articles were calculated. These totals were used to compute the proportion of articles dedicated to the topic of aging in place over time. Retrieved articles were excluded from analyses if their contents were book or audiovisual reviews, conference abstracts, or editorials. Finally, we used the advanced search option on each journal's official website, where possible, to identify articles containing any of the three exact phrases and their variations in titles, abstracts, key words, or in the bodies of articles—articles were not counted in this step if key terms appeared only in the bibliographies of papers. For journal websites without this advanced search option, the advanced search option under Google Scholar was used for searching within the specific journal. A similar approach was used by Carr and colleagues [7] in their review of arts and aging research. 

Articles that were retained were coded according to three criteria. First, articles were sorted according to whether their content dealt directly with the concept of aging in place (direct) or whether key terms were mentioned only in passing in articles primarily about other topics (indirect). Next, we indicated whether aging in place articles were focused on five areas that influence the capacity of older adults to age in place—these included subcategories for housing/environmental considerations (e.g., neighborhood characteristics and home modifications); community/social services (e.g., influence of church groups, barriers to access of services); assistive devices/technology (e.g., telemedicine, remote assessments, and silver alerts); health and functioning (e.g., supportive housing for frail adults); a miscellaneous category that contained factors that did not fit into the other four subcategories, such as issues pertaining to migratory patterns or municipal zoning regulations. Any single article could be categorized in one or more of these topic areas, depending on the range of scope of the article. Finally, we sorted articles by whether the content was primarily research oriented, or whether articles discussed policy pertaining to aging in place. Articles were categorized as research oriented if concepts of aging in place were analyzed empirically as an antecedent (an independent variable), a mediator (a process variable), or an outcome (a dependent variable). Articles were categorized as policy oriented if their content discussed program development or implementation of programs where aging in place was a stated goal. 

The search and review of manuscripts were conducted by two readers (SV and BAS), who determined the criteria for inclusion and categorization of manuscripts prior to review. Each reader independently reviewed the possible manuscripts. When the readers disagreed on the categorization of any article, disparate cases were discussed and an agreed upon consensus for classification was established and recorded, before data were analyzed. We conducted frequency analyses and computed the proportion of aging in place articles relative to the total articles published during the period of interest. We also conducted frequency analyses differentiated by whether articles mentioned aging in place as an indirect concept, or whether aging in place was the central issue discussed by the article (direct concept). Finally, we calculated the frequency of aging in place articles by subcategory topic (i.e., whether articles addressed housing, services, technology, health, and/or some other topics), and by whether articles were research focused or policy focused.

## 3. Results

**Figure 1:**
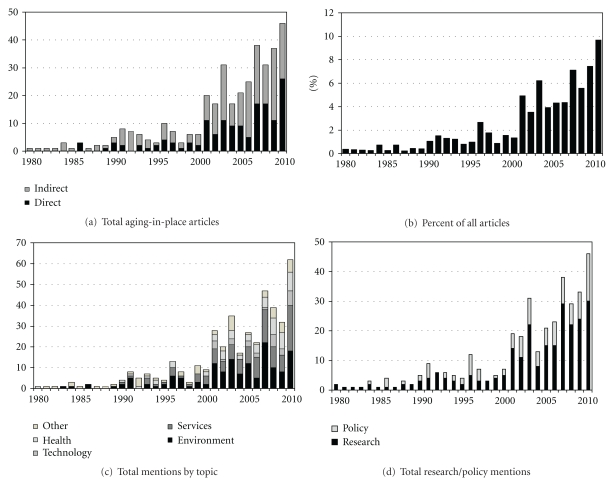
(a) Absolute number of aging-in-place articles (direct and indirect mention), 1980–2010; (b) proportion of aging-in-place articles relative to the total published articles, 1980–2010; (c) absolute number of categorical mentions among aging in place articles published, 1980–2010*; (d) total aging in place articles by research/policy designation. *Categorical mentions are not mutually exclusive (e.g., one manuscript may have multiple category mentions).

Among the journals examined, there was an increase in the publication of aging in place manuscripts from 1980 to 2010 (Figure 1(a)). During the 1980s, very few publications included this concept. The number of these articles nearly doubled in the 1990s, and a marked increase in the absolute number of manuscripts pertaining to aging in place began in 2001, with the highest number of publications in the most recent year of 2010. The initial inclusion of “aging in place” in the literature (1980s) generally mentioned this concept indirectly, and it became a central part of some published articles in the 1990s, where the ratio of direct to indirect mention was about 0.55. From 2000 to 2010, this ratio of direct to indirect mentions increased to 0.75, with an excess of direct mentions relative to indirect mentions published in 2001 (ratio: 1.22). During the overall 1980–2010 period, nearly 70% of all aging in place manuscripts indirectly mentioned this concept. 

When we considered the number of aging in place publications relative to the number of overall journal publications (Figure 1(b)), the trends over time were remarkably similar to the absolute number of aging in place manuscripts (Figure 1(a)). This suggests that the proportion of aging in place articles has increased over time. Aging in place articles have also expanded in the diversity of the topics covered (Figure 1(c)) from 1980 to 2010. In the earliest decade (1980–1989), environment and the “other” category (including mostly articles pertaining to migration) dominated the aging-in-place literature. Over the following ten years (1990s), aging in place manuscripts extended to areas of service, and there was some mention of health and functioning. During this time, topics related to the environment remained a leading area of focus for aging in place publications. The 2000s marked a time of increased breadth of topics covered among the aging-in-place literature. The topic of the environment and services were the most commonly examined areas during the period 2000–2010 (35% and 31%, resp.), with 15% of the articles pertaining to health and functioning and 10% representing the “other” category. Articles related to technology became more prominent during the 2000s, representing 9% of aging-in-place publications. When we examined the entire 1980–2010 time frame, this trend resembled that of the lattermost decade: environment (36%), services (29%), health and functioning (15%), other (13%), and technology (7%).

Upon classifying articles as empirical research based and/or explicitly pertaining to or mentioning policy, we noted an increasing absolute number of both empirical and policy-related articles over time (Figure 1(d)). Interestingly, the proportion of research-based to policy-related articles markedly increased between the 1990–1999 and 2000–2010 period. During 1990–1999, research articles were nearly 1.5 times as prevalent as policy-related manuscripts. This ratio increased over the 2000–2010 timeframe, where the proportion of research to policy articles on aging in place was 2.5.

Our initial efforts to determine how aging in place has developed over time and across topics have yielded a number of important points. Of note in our analyses was the increase over time of the “other” category, which was comprised mostly of issues surrounding older adult migration between regions in the US, concerns surrounding older immigrant adults, and municipal-level factors, such as zoning regulations. In addition, we noted four other important trends in the literature that affect the ability of older people to age in place. First, aging in place publications span a wide spectrum ranging from broad to specific investigations. Some broad depictions of manuscripts discuss this concept within the context of the worldwide greying of our communities [8] and exploratory, qualitative analyses (e.g., determining the amenities that individuals currently utilize to age in place [9]). The more specific papers on aging in place focus on services (e.g., nursing homes and assisted living facilities [2], health monitoring [10], housing and social support [11, 12], and palliative care [13]).

Second, with respect to the environment, aging in place has two prongs: aging in place in the home and in other structured settings in the community. While the definition of “home” varies (e.g., single or multiple family home) and continues to remain an essential component of aging in place, increasing attention has highlighted the importance of community care as a means to either support aging in place or as an initial step in fostering the goal of aging at home [14, 15]. 

Third, aging in place is not a one-size-fits-all concept. There are multiple issues surrounding differences in aging in place among diverse populations. Such diversity arises from differences in preferences and access to services with regards to differences in rural versus urban settings [16, 17], income [18], orientation (e.g., lesbian, gay, bisexual, and transgender sensitivities [19]), older adults with special needs (e.g., intellectual disabilities [20] and prisoners [21]), older adults with special circumstances (e.g., adults who are caring for children with developmental disorders [22]), more general differences in eastern versus western views on aging in place [23], and broad international differences in services that individuals require, want, and need [24]. Regardless of these differences, the concept of aging in place has established itself internationally, with studies documented in Sweden [25], China [26], the United Kingdom [27], Japan [28], New Zealand [29], Australia [30], Malaysia [31], and Taiwan [32].

Fourth, technology has become an increasingly important component to the literature on aging in place. The worker interactive networking project is an example of the growing number of studies that examine the influence of technology in supporting working-family caregivers of frail and memory-impaired older adults [33]. Other studies focus on the mobile and e-communications among older Japanese adults [28], telecare initiatives to address issues related to the potential negative experiences associated with aging in place (e.g., lack of informal support [27]), and pain management through videoconferencing [34]. 

Although a number of articles focus on the importance of aging in place [35], others highlight the potentially negative experiences (e.g., isolation and loneliness) associated with remaining in the same location [27]. Such ideas are echoed by LeRoy and colleagues [36] who cautioned that aging in place does not assure a high quality of life, since continuity of place is not always accompanied by a continuity of roles, relationships, and lifestyles (often the case for adults with dementia). Further evidence for this is provided by reports that changes in the environment can be associated with positive outcomes [37], in which older adults relocate to enhance individual development, pursue personal interests, and overcome restrictive environments.

## 4. Discussion

This study documents the increasing attention given to aging in place in the gerontological research community over the past 30 years. Our findings indicate the growing variety of topics pertaining to aging in place, ranging from housing and environment to health and technology. The relevance of this topic, we believe, has increased over time, in part due to the acknowledged preference of older persons (and younger persons with disabilities) to maintain independence, and to the greater availability of noninstitutional care. In addition, concerns about the escalating costs of institutional long-term care on the part of policy makers have made a priority of concerns by older persons and their families, regarding the desire to avoid relocation in order to receive needed assistance. As a result of this reprioritization, new grants have been initiated to foster aging in place efforts that are based on evidence-based research findings, under the auspices of the National Institute on Aging (NIA), the Administration on Aging (AoA), and other federal agencies.

Although we believe that our findings illuminate a real and important growth in the quantity and diversity of aging-in-place publications, we acknowledge some limitations of the current study. Namely, by including only academic manuscripts in the eleven designated gerontology journals, we excluded books, scholarly publications from other related journals (e.g., those specifically pertaining to housing, economics, and technology), and reports by organizations that have focused specific attention on this issue (e.g., AARP). Our selected search terms also limited the inclusion of some publications, because of different terminology used among countries and cultures. For instance, some Europeans often use the term “staying put,” while other articles have used “home independence” to encompass the concept of aging-in-place. The use of the selected search terms to study aging in place would represent a more conservative estimate of trends and may provide a selective perspective of the concept. The current study provides a general synopsis of the trends in aging in place literature from 1980 to 2010, but further studies that examine this body of work across a number of other categories are warranted (e.g., studies that are classified based on cross-sectional versus longitudinal methods, interventions, personality, and subjective/objective perceptions regarding aging in place). Despite these limitations, we believe our analyses illustrate the emergence and arrival of aging in place as a focal concept in the scholarly field of gerontology. 

In conjunction with worldwide population aging and the greater likelihood of surviving to an age when individuals are likely to require some form of daily assistance to achieve independent living, we have documented the concurrent growth in attention paid by gerontologists who often influence policy decisions regarding strategies and barriers to aging at home. Unfortunately, as of 2011, many barriers remain for older adults seeking alternatives to institutional care. This includs limited funding for programs that provide home modifications, service delivery issues, consumer awareness and training issues, and poor communication among government agencies that address health, housing, and services for older adults and people with disabilities [38]. In addition, excess expenditures associated with aging in place may, at times, outweigh alternative options to age in other settings [39]. Conversely, the savings overall, associated with multiple noninstitutional alternatives, may not always accrue to any particular program that provides support for aging in place. Aging in place may also require much more involvement of relatives, friends, and unpaid community members than involvement of institutional settings.

As a result of these challenges, there is a continued need for research and policy development that can be applied to address these problems. Specifically, researchers should continue to explore how policies, services, environment, and technology influence aging in place, as well as the degree to which aging in place research informs and influences policy and services. Perhaps the biggest question surrounding our results pertains to how trends in aging-in-place literature translate to the needs and services currently provided to older adults. Additional studies are warranted in order to address this important and pressing question. Although aging in place seems to have come of age over this 30-year time period, we expect that future trends will exhibit a greater diversity of aging-in-place topics and that this concept will continue its upward trajectory within gerontology publications.
